# High-Accuracy Chicken Breed Identification Using Microsatellite Genotype Data and AutoGluon Framework

**DOI:** 10.3390/biology15010021

**Published:** 2025-12-22

**Authors:** Rajaonarison Faniriharisoa Maxime Toky, Sutthisak Sukhamsri, Sadeep Medhasi, Trifan Budi, Thitipong Panthum, Worapong Singchat, Kornsorn Srikulnath

**Affiliations:** 1 Animal Genomics and Bioresource Research Unit (AGB Research Unit), Faculty of Science, Kasetsart University, Bangkok 10900, Thailand; rajaonarison.t@ku.th (R.F.M.T.); sadeep.m@chula.ac.th (S.M.); trifan.bu@ku.th (T.B.); thitipong.pant@ku.ac.th (T.P.); worapong.singc@ku.ac.th (W.S.); 2 Department of Information Technology, Faculty of Science and Agricultural Technology, Rajamangala University of Technology Lanna Tak, Tak 63000, Thailand; 3 Biodiversity Center, Kasetsart University (BDCKU), Bangkok 10900, Thailand

**Keywords:** breed determination, chicken breeds, machine learning, microsatellite, random forest

## Abstract

The practical applications of breed identification are numerous and diverse, and they include breed conservation and breeding program design. However, distinguishing between breeds remains challenging and costly, especially for phenotypically similar chicken populations. Continued research is necessary to develop more accessible and optimized methodologies. To address these challenges, machine learning (ML) offers promising tools for analyzing complex genetic data. The capabilities of machine learning, especially the random forest (RF) model, to enhance various fields, including bioinformatics, have recently been demonstrated. In this study, microsatellite genotype data from 651 individuals across 30 chicken populations filtered from a larger initial dataset for consistency were used to classify breeds using an RF model. Cross-validation techniques, including 10-fold cross-validation and leave-one-out cross-validation, were employed to assess the performance of the model. The model performance was evaluated using metrics such as accuracy, Cohen’s Kappa, 95% confidence interval, and F1-score. Results showed that the RF model achieved a 95.38% accuracy on the testing dataset. Accuracies of 91.44% and 90.99% were observed for 10-fold cross-validation and leave-one-out cross-validation, respectively. It is believed that larger datasets will significantly improve outcomes for other breeds. Because of its generalizability, the trained model can serve as a straightforward and modern method for chicken breed determination using machine learning. This study demonstrates that ML, particularly automated approaches like AutoGluon, provides a robust and accessible framework for chicken breed identification using cost-effective microsatellite data.

## Simple Summary

Identifying chicken breeds correctly is important for conserving local breeds and improving breeding programs. However, many breeds look very similar, making visual identification difficult and sometimes inaccurate. In this study, genetic information from several chicken populations was used to train a machine learning model to recognize breed patterns. The model was tested and showed high accuracy in identifying most breeds. This demonstrates that computer-based methods can offer a practical and reliable tool for farmers, breeders, and conservation groups. As more genetic data becomes available, this approach is expected to become even more accurate and useful for protecting and managing valuable chicken breeds.

## 1. Introduction

Chicken breeds represent important global genetic resources for food security, cultural heritage, and environmental resilience, yet conserving their genetic diversity remains a major challenge worldwide [1,2]. Breed identification involves classifying an animal into a group characterized by a homogeneous phenotype. Accurate classification is vital in animal breeding, including livestock, poultry, and aquaculture, as it forms the basis for maintaining specific traits and performing essential operations such as selective breeding and resource management [1,2,3]. Various approaches, including morphological identification and molecular markers, have been developed for breed identification or confirmation [4,5,6,7]. Molecular marker identification involves methods such as Amplified Fragment Length Polymorphism (AFLP), microsatellite genotyping, Single nucleotide polymorphism (SNP) panels with microarray, and whole-genome sequencing. However, most of these approaches are costly, time-consuming, require advanced genomic expertise, and involve labor-intensive processes. For instance, SNP panels with microarrays and whole-genome sequencing are highly informative for breed identification owing to their examination of many genetic loci. However, their high cost makes them impractical for use in laboratories or communities in developing countries (Global South). Additionally, farmers and breeders, who benefit most from breed identification, often lack the necessary experience, making expense reduction a critical concern [8,9]. Legacy methods for breed identification remain difficult to access and costly for many users. Some uncertainties regarding the origins of certain chicken breeds, particularly when phenotypic similarity is involved, have recently been clarified [10]. Improved methods are needed to facilitate this process.

Microsatellite DNA, or short tandem repeats, are short, repetitive sequences of 1–6 base pairs that are tandemly repeated in the genomes of both prokaryotes and eukaryotes [11]. Owing to their co-dominant inheritance, high allelic diversity per locus—referred to as high multiplex ratio—and the ease and cost-effectiveness of amplification by PCR, microsatellites are widely used as genetic markers across all continents [12,13,14]. Prior research has indicated that these tandem repeats are closely associated with the genetic structure of populations [15]. The strong correlation between microsatellite markers and breed specificity makes them valuable tools for various genetic studies, including breed differentiation in animals, particularly chickens [16,17,18,19]. Genotypic data for microsatellite markers, which represent allele information at specific loci, are obtained by extracting DNA, amplifying microsatellite regions via PCR, and determining allelic sizes [11]. The genetic variation between individuals is organized into a genotypic data table for further analysis. When the genotypic data are large and sufficient, representing multiple breeds and localities, the accuracy of breed identification is strengthened. The Siamese chicken bioresource project, as described by Wattanadilokchatkun et al. [20], provides microsatellite genotype data across many chicken populations and breeds, serving as a valuable resource for breed identification. However, determining breeds using genotypic data involves clustering analysis and probability, which sometimes results in chickens from the same breed not being completely grouped but overlapping with other breeds. This may be due to high genetic variation within breeds. Developing methods to justify and confirm breed identities is necessary to improve reliability and practicality.

Over the past decade, advances in artificial intelligence and increased computational power have made machine learning (ML) widely accessible and effective for analyzing complex biological data [7]. The choice of ML algorithms depends on the study objectives and data structure, with supervised learning being particularly suitable when labeled data are available [21,22,23]. In this context, Random Forest (RF) is well-suited for genotypic data because it can handle high-dimensional, categorical predictors and capture complex interactions among loci without requiring assumptions about data distribution. These properties make RF a robust approach for genotype-based classification tasks. Two main categories of supervised models are distinguished: regression models and classification models [24]. The most notable supervised learning algorithms include decision trees, K-nearest neighbors (KNN), support vector machines (SVM), and random forests (RF). In bioinformatics, various ML algorithms have been adopted to address complex classification tasks involving high-dimensional genetic data. Commonly used models include decision trees, SVM, KNN, ensemble methods such as RF, and gradient boosting frameworks like LightGBM and XGBoost. Recently, autoML platforms such as AutoGluon have emerged, offering streamlined approaches that combine multiple models and optimize hyperparameters [25,26]. Of these methods, the RF model has become a popular choice owing to its efficiency and high predictive accuracy across various data types, including those with large attribute spaces and complex structures [27,28]. The RF model, which was initially proposed by Breiman (2000, 2001, 2004) [29,30,31,32], can be applied to both regression and classification tasks, and is effective in a variety of practical applications [33,34,35,36]. The RF algorithm, which operates by constructing multiple decision trees, trains each tree independently to classify data instances, and the final classification is determined by majority vote across all trees [37]. This ensemble approach, which is used to enhance predictive accuracy and generalizability, is considered superior to single models or the decision tree [38,39].

Although microsatellite genotyping has been widely used in population genetics and breed characterization in various animal species, its application in ML-based classification of chicken breeds remains underexplored. Burócziová and Říha (2009) [40] provided an overview of the use of classification algorithms with microsatellite genotype data to identify genetic differences between horse breeds. However, few studies have systematically explored whether similar approaches can be applied to chicken breeds, particularly using ensemble models like RF. It is expected that using these genotype data to train RF models will achieve high performance in chicken breed prediction. Genotype data from multiple chicken populations were compiled and used to train RF-based classification models. These models were assessed with robust cross-validation techniques to ensure reliability, supporting the development of a more accessible, scalable, and accurate breed identification system with applications in conservation and breeding.

Therefore, this study aims to evaluate the performance of a Random Forest model and the AutoGluon—AutoML framework for classifying chicken breeds using microsatellite genotype data, providing a comparative analysis of their accuracy and practicality.

## 2. Materials and Methods

### 2.1. Microsatellite Marker Genotype Dataset

Data on chicken genotypes used in this study were obtained from https://doi.org/10.5061/dryad.hhmgqnkm0 (accessed on 6 June 2024) [20] and “[Dataset] Supplementary Information: High-accuracy chicken breed identification using microsatellite genotype data and AutoGluon framework by Toky et al.” from the Kasetsart University Knowledge Repository (https://kukr.lib.ku.ac.th/kukr_es/dataset, accessed on 22 December 2025), comprising 651 individuals across 30 populations (11 indigenous chicken breeds, 3 local chicken breeds, and 16 populations of red junglefowl), which were also under the Siam Chicken Bioresource Project (https://www.sci.ku.ac.th/scbp/; accessed on 30 June 2024) (Table S1) [10,20,41,42]. The genotype matrix was composed of 28 bi-allelic microsatellite loci. Twenty-eight microsatellite primer sets were selected based on the recommendations of the Food and Agriculture Organization for chicken biodiversity assessments [43]. To ensure robust model performance, populations with fewer than 30 individuals were excluded from the analysis; however, the number of training samples was sufficient, even after data splitting for testing purposes. The data was initially formatted using Microsoft Excel, after which the genotype data were imported into a Pandas DataFrame using Pandas version 2.2.2, and a manual class weighting strategy was implemented to address class imbalance during model training [44]. This was achieved by using the compute_class_weight function from the sklearn.utils.class_weight module, which ensured appropriate weighting of classes with fewer samples to mitigate potential bias during training. A comprehensive list of the populations included in the final analysis is provided in Table 1. The proportion of data reserved for testing was optimized through experimentation with various splits, ranging from 10% to 30%. The optimal testing split was identified as 15%, which produced the best-performing model. Feature scaling was also applied to both training and testing datasets to minimize inaccuracies in predictions. This scaling, which prevented larger magnitude datasets from disproportionately influencing the results, was achieved using StandardScaler from the scikit-learn library version 1.5.1 [45,46]. The RF model was used as the baseline, while the AutoGluon framework, which incorporated hyperparameter tuning across various models, including ensemble methods, tree-based algorithms, linear models, neural networks, and KNN, was applied to enhance predictive performance for chicken breed classification.

### 2.2. Baseline Model: RF Model

Training the model: The models were developed and evaluated using Python 3.12.4 programming language, which was installed via the Conda 24.5.0 package on the SUSE Linux Enterprise Server 15 SP4 operating system. All the requisite Python modules were incorporated into the environment to facilitate this analysis [47,48]. Both linear and non-linear feedforward neural networks were tested for multiclass classification tasks using PyTorch version 2.3.1.post100 [49,50]. The neural network architectures were constructed using the nn submodule from the primary PyTorch package, torch. In the case of non-linear models, the hidden layers were configured to use the rectified linear unit activation function, thereby introducing non-linearity. By contrast, the linear models used solely the nn.Linear class. A variety of combinations of hyperparameters, optimizers, loss functions, and training epochs were investigated.

Preliminary Model Comparisons: The neural network models, however, attained a maximum accuracy of 73% on this dataset. Random Forest (RF) was selected as a baseline classification model because the dataset comprised multiple categorical and mixed-type predictors rather than a single continuous variable. RF effectively captures complex interactions among categorical variables through an ensemble of decision trees trained on different subsets of samples and features [31]. The RF model was implemented using the RandomForestClassifier class from the scikit-learn.ensemble module of scikit-learn version 1.5.1, and an accuracy of 75.51% was achieved with the use of only 15 decision trees, indicating slightly superior performance [51,52]. The performance of the RF model was further optimized by conducting experiments with varying numbers of decision trees. Since the RF algorithm can handle categorical data, the two allelic values at each bi-allelic locus were concatenated using an underscore as a separator [53,54]. This approach ensured that the model interpreted the data as distinct categorical combinations rather than as a single continuous value, while robust performance was guaranteed using a previously described class weighting [55]. After comparisons against Gini, the criterion for determining the optimal splits at each node was set to entropy, calculated using Equation (1):

(1) $$Entropy\left(S\right)=-\sum_{1=1}^{C}p_{i}\;{log}_{2}\left(p_{i}\right)\;$$

where *p_i_* represents the proportion of class I in the subset, and C is the number of classes. To ensure training reproducibility and consistency, the random_state was fixed at the arbitrary value of 42, which guaranteed the reproducibility of random processes, including data and feature subset selection for each decision tree, as described by Liaw and Wiener (2002) [53] and Breiman (2001) [31].

The performance and behavior of ML models are significantly influenced by the hyperparameters used in their configuration [56]. Proper tuning of these parameters is crucial, as it can significantly enhance the generalizability of the model to unseen data. To optimize the hyperparameters of the model, a tuning procedure was conducted using the RandomizedSearchCV function from the sklearn.model_selection module, which involved exploring a range of hyperparameter configurations through a randomized search of five distinct random combinations of parameters. The number of decision trees in RF was varied from 10 to 1000, with the maximum depth of each tree set to “None,” allowing for unrestricted growth. The search was executed over 50 iterations, during which a three-fold cross-validation strategy was used to assess the performance of each hyperparameter combination. This process was undertaken to identify the optimal hyperparameters that maximize the accuracy of the model

Model evaluation using cross-validation techniques: The model performance was evaluated using two cross-validation methods, repeated 10-fold and leave-one-out, which provided a comprehensive understanding of its robustness and reliability [47,57]. In repeated 10-fold cross-validation (R10FCV), the dataset was divided into ten equal partitions, or folds. The model was trained on nine of the folds and tested on the remaining fold. This process was repeated ten times, with each fold serving as a test set once. The performance metrics were subsequently averaged across the ten iterations to obtain the total estimate of the model performance. With the leave-one-out cross-validation (LOOCV), each sample in the dataset was used as a test set once, with the remaining n−1 samples used for training. This approach results in n iterations, where n is the total number of samples. This ensures that each sample is tested individually while the model is trained on all other samples. The model is trained and evaluated on each data point of the dataset. This approach enables a comprehensive evaluation of the model, although high computational costs are incurred for large datasets. R10FCV was implemented using the StratifiedKFold class from the model_selection module, with n_splits set to 10 (k = 10). This ensured that each fold was representative of the overall class distribution. LOOCV was performed using the LeaveOneOut class, which provided a straightforward method for performing this type of cross-validation. These cross-validation techniques provided insights into the generalizability of the model to unseen data.

Model testing and performance assessments: In addition to cross-validation analyses, the predictive performance of the model was evaluated on manually defined test splits (85% training and 15% testing) using the “predict” method of the trained classifier instance, which generated predictions based on the test data. The precision of these predictions was evaluated using the torch.eq function, which performed an element-wise comparison between the predicted values and true labels, resulting in a tensor that indicated where the predictions matched the true labels (Figure S1). The accuracy of the model was then calculated based on the proportion of correct predictions relative to the total number of predictions as follows:

(2) $$Accuracy=\left(\frac{Correct\;predictions}{Total\;number\;of\;predictions}\right)\times 100$$

In evaluating classification models, the term “agreement” describes the degree to which two outputs produce the same classification. The Cohen’s Kappa metric was employed to assess the agreement between predicted and true classifications, accounting for the chance agreement that may occur [58]. Kappa’s value is calculated as follows:

(3) $$K=\frac{P\left(A\right)-P(E)}{1-P(E)}$$

where P(A) is the observed agreement and P(E) is the expected agreement by chance. It provides a measure of the improvement in model performance relative to what might be expected by chance alone, thus offering a more comprehensive understanding of model performance [59].

For each class, precision is defined as the ratio of true positives (TP) to the sum of TP and false positives (FP), while recall is the ratio of TP to the sum of TP and false negatives (FN) [60,61]. TP are correct classifications, FP are incorrect classifications to other classes, and FN are instances belonging to other classes but predicted to be among the current considered class. Precision and recall are calculated using Equations (4) and (5), respectively:

(4) $$Precision=\;\;\;\;\;\;\;\frac{TP}{TP+FP}$$

(5) $$Recall=\;\;\;\;\;\;\frac{TP}{TP+FN}$$

The harmonic means of precision and recall, also known as the F1-score, is calculated using the following equation:

(6) $$F1\;Score=\;\;\;\;\;\;2\times \frac{Precision\times Recall}{Precision+Recall}$$

The F1-score serves as a critical metric for assessing model performance, particularly in scenarios involving class imbalances [62]. As the F1-score provides a comprehensive understanding of TP, FP, and FN, it is essential in classification projects where false positives and false negatives can have serious consequences [63,64].

### 2.3. Optimized Model: AutoGluon Approach

**Figure 1:**
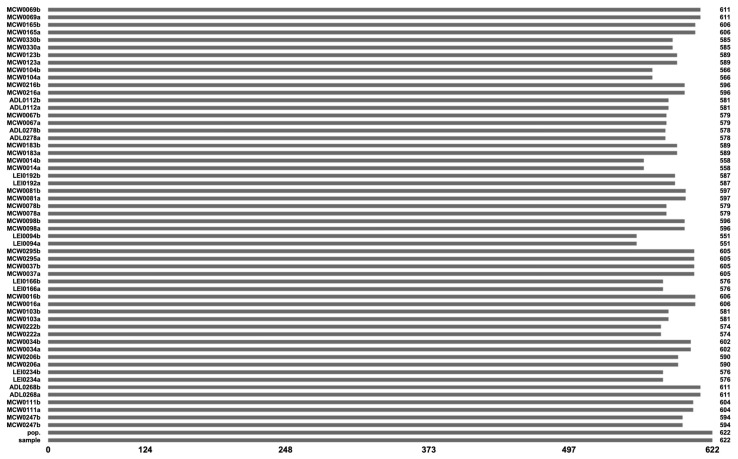
Visualization of nullity by column of the dataset.

The methodological workflow began with data preprocessing to ensure data quality and balance. Missing values within the dataset were addressed using the MISSINGNO technique, which provides a systematic visualization and imputation to handle incomplete entries. To address class imbalance, particularly prevalent in categorical classification tasks, the synthetic minority oversampling technique (SMOTE) method was applied to generate synthetic samples for minority classes, thus enhancing the representativeness of the training data with an AutoGluon approach in the final step (Figure 1).

Machine-learning data preprocessing: The dataset was composed of genetic data collected from various chicken breeds to facilitate breed classification using ML techniques. It included 622 samples and 58 columns, which consisted of 56 genetic marker parameters, a unique identifier that was labeled “sample,” and a target column that was labeled “pop”. To ensure a comprehensive understanding of data quality before further processing, a thorough examination of missing values was conducted. The MISSINGNO tool was used to generate a visual overview of the distribution of missing data, which enabled the identification of features or samples with incomplete information. Following the results obtained from the MISSINGNO library, varying degrees of missing data were observed across different genetic markers in the dataset. The proportion of missing values ranged from approximately 1.7% to 11.4%, with a significant concentration found in genetic markers belonging to the locus-specific extended information (LEI) sequence group. This finding shows that certain microsatellite markers within the LEI sequence exhibited a higher tendency for incomplete data compared to other genetic markers, such as Microsatellite Chicken Washington (MCW) or avian disease and leukosis (ADL) sequences. Notably, lower levels of missing data were displayed by MCW and ADL markers, indicating their robustness in genetic studies. To ensure that missing data does not introduce bias or negatively affect ML models, it is essential to address these gaps to maintain dataset reliability, improve data preprocessing steps, and ensure robust breed classification predictions based on genetic markers. To handle missing values in the dataset, the KNN imputation method was employed. Missing values were estimated based on the similarity between samples, with K samples identified as the most similar to the sample with missing data using a selected distance metric, such as Euclidean distance. The missing values were then replaced with the average numerical values from the nearest neighbors, ensuring that the imputed values preserved the genetic structure and patterns within the dataset.

The optimization of the KNN imputation process involved identifying the optimal number of *K* samples with the smallest genetic distance, which were used to estimate the missing data. The selection of *K* is crucial, as a smaller *K* can lead to overfitting, while a larger *K* can cause excessive smoothing, thereby reducing the variability of the dataset. The KNN imputation method estimates the missing value ${\hat{x}}_{i,j}$ using the values of the same feature from the KNN of the sample. The *K* = 3 value was chosen as it was considered optimal for data imputation. The imputed value ${\hat{x}}_{i,j}$ is computed as follows:

(7) $${\hat{x}}_{i,j}=\frac{1}{k}\sum_{m=1}^{k}x_{m,j}$$

where $x_{m,j}$ is the value of the *j*-th feature for the *m*-th nearest neighbor and *k*: is the number of nearest neighbors to consider.

**Figure 2:**
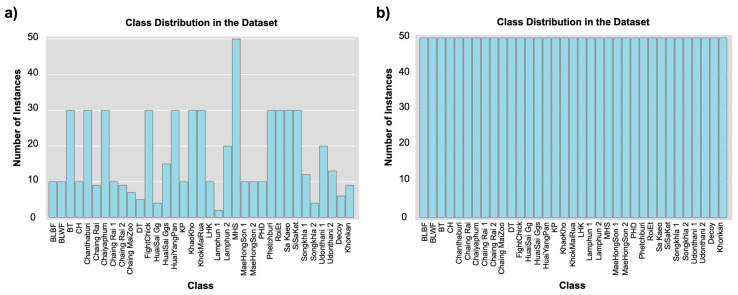
Class distribution before and after dataset balancing. (**a**) Original dataset showing unequal sample counts across chicken breed classes. (**b**) Balanced dataset after applying the Synthetic Minority Over-sampling Technique (SMOTE), where class sizes were standardized to approximately 50 instances per class.

Following the imputation of missing data, attention was directed toward class imbalance in the target variable. In classification tasks, the target variable is often highly skewed, as shown in the class distribution (Figure 2), which visually represents the number of instances available for each chicken breed in the dataset.

A closer examination revealed that the dataset exhibited significant class imbalance, which could affect the performance of any predictive model applied to it. Certain classes, such as “MHS,” contained a disproportionately high number of instances, while other breeds, including “Lamphun_1” and “HuauSai_Gg,” had significantly fewer samples. This disparity may lead to biased ML models that favor well-represented breeds and underperform on underrepresented classes, distorting the predictive accuracy. To ensure unbiased data before developing classification models, a well-balanced dataset is essential. Data imbalance may result in overfitting in dominant classes, where patterns are primarily learned from breeds with abundant data, while those with fewer samples are overlooked. To mitigate this issue, SMOTE was used to synthesize the imbalanced data. This method generated synthetic samples for the minority class by interpolating between existing minority class samples instead of duplicating existing ones. For a given minority class sample $x_{i}$, SMOTE selects one of its KNN $x_{zi}$ and generates a new synthetic sample $x_{new}$ as follows:

(8) $$x_{new}=x_{i}+\lambda \cdot \left(x_{zi}-x_{i}\right)\;\;$$

where

$x_{i}$: A minority class sample.

$x_{zi}$: One of the K-nearest neighbors of $x_{i}$

λ: A random number between 0 and 1, which determines the position of the new sample along the line segment connecting $x_{i}$ and $x_{zi}$.

**Figure 3:**
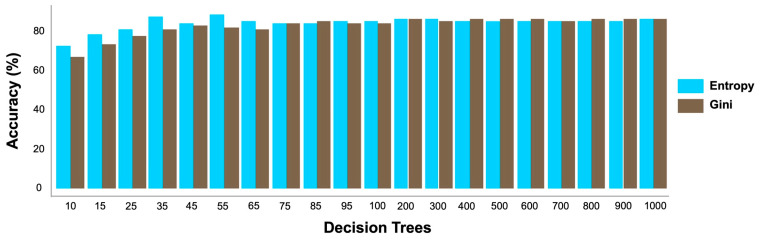
Performance testing of the random forest model on the whole microsatellite genotype chicken dataset with 30 populations. Evolution following the increase of decision trees. Comparison of entropy and Gini functions as criteria to define data split in trees.

The result of applying SMOTE to the dataset was oversampling, which led to a balanced distribution with 50 instances in each class (Figure 3). The final step in data preprocessing was feature standardization, which is critical for ensuring that the data is in an optimal format for training ML models.

One of the most used techniques for standardization is the StandardScaler, which is a powerful tool in ML and data analysis. The features of the dataset were transformed using the StandardScaler to have a mean of 0 and a standard deviation of 1, which was achieved by subtracting the mean of each feature from the data points and dividing by the standard deviation. Mathematically, this transformation is represented as

(9) $$z=\frac{x-\mu }{\sigma }\;$$

where x is the original feature value, μ is the mean of the feature, *σ* is the standard deviation of the feature, and *z* is the standardized feature value.

This standardization process ensured that all features were centered around zero and had a consistent scale, which is particularly important for ML algorithms that are sensitive to the magnitude of input data. Without standardization, features with larger ranges could dominate the learning process, leading to biased or suboptimal model performance.

### 2.4. Optimized Model: AutoGluon

After data preprocessing, the model was developed using the AutoGluon framework, which automates the entire ML. This framework generates and selects features and identifies the most relevant attributes for the classification task. It trains a diverse set of base models, including tree-based algorithms (LightGBM, XGBoost, CatBoost), linear models, KNN, and neural networks, applying automatic hyperparameter tuning and cross-validation to ensure robust performance. Following the base-model training, AutoGluon used a multilayer stacking ensemble strategy, combining predictions from multiple models through meta-models to improve generalization and reduce overfitting. The final model was selected based on the F1-score, which is especially suitable for imbalanced classification tasks.

The analysis pipeline, code, and hyperparameters of the final optimized models are available in the Supplementary Information.

## 3. Results

The RF model was tested on microsatellite genotype data of chicken breeds and demonstrated superior performance compared to other evaluated models, including linear and non-linear feedforward neural networks, KNN, and ensemble models implemented via the AutoGluon framework. The model was initially configured with only the number of decision trees and impurity criterion, using the complete unfiltered dataset. The number of trees was varied from 10 to 1000, and an accuracy of 75.51% was achieved with as few as 15 trees (Figure 3). A notable increase in accuracy was observed up to 55 trees, after which performance plateaued at around 81%. Entropy performed better than the Gini index, particularly between 10 and 65 trees, and was therefore selected as the impurity measure (Figure 3), which showed bootstrap sampling and node generation based on minimum entropy. Populations with fewer than 30 individuals were then excluded to ensure sufficient training data; consequently, 13 chicken populations comprising 433 individuals were used to tune the model to optimize performance.

### 3.1. Performance Evaluation of the Hyperparameter-Tuned Random Forest Model

Hyperparameters were tuned using 85% of the training data to optimize the performance of the RF model. During tuning, the number of cross-validation folds was varied from 2 to 9, with the best model performance obtained when 3 samples were included per fold. Tuning the hyperparameters of the filtered dataset resulted in an accuracy of 95.38%, which was a significant increase from that of the baseline model. Both models were evaluated using 15% of the testing genotype data. The trained model was used to estimate membership probabilities for each class through a voting mechanism (Table 2). In the training phase, each decision tree was built from a randomly selected subset of the training data. For every test sample, predictions were generated by all trees, which assigned probabilities for class membership. The final prediction was based on the average of these probabilities, with the class receiving the highest average being selected (Table 2). This approach helped reduce overfitting and increased model robustness, and ensured that the final output was the most consistent and probable among the ensemble. A significant disparity was noted between the achieved accuracy (95.38%, Table 3) and the No-Information Rate (16.92%), which underscored the ability of the model to learn from the data. Even the lower bound of the 95% confidence interval (CI = 0.9028, Table 3) reflected strong performance in the worst-case scenario. The Cohen’s Kappa value was 0.9492, which shows a high level of agreement between predicted and actual classifications, affirming the reliability of the model beyond random chance (Table 3). The confusion matrix further evidenced the performance of the model, with most values aligned along the diagonal and few off-diagonal errors, indicating minimal misclassifications (Figure 2). This distribution confirmed the robustness of the model in distinguishing between chicken breeds. The F1-score, which represented the harmonic mean of precision and recall, was calculated for each class. Eight out of thirteen classes achieved an F1-score of 1.00, while two classes (“Petch Ggs” = 0.67 and “KMR Ggg” = 0.75) received lower scores, resulting in a macro average of 0.93, which highlighted the overall effectiveness of the classification model (Table 4).

### 3.2. Cross-Validation Results

A further evaluation of the performance of the RF model in classifying chicken breeds was conducted using cross-validation techniques. The overall accuracies and Kappa values facilitated a direct comparison between R10FCV and LOOCV (Table 3). The principal difference between these methods was in the manner of testing the splits. LOOCV used one sample per fold, whereas R10FCV used k = 10 samples per fold. Both methods were effective as high model performance was consistently achieved on the chicken genotype dataset, with accuracies significantly exceeding the No-Information Rate of 16.17% (Table 3). This performance showed that the RF model can learn meaningful patterns from the data, regardless of the validation method used [65]. Kappa values of 0.9065 for R10FCV and 0.9016 for LOOCV were obtained, indicating that reliable classifications were made well beyond random chance. Corresponding accuracies of 91.44% and 90.99% for R10FCV and LOOCV, respectively, further validated the effectiveness of the model. R10FCV was slightly more effective (Table 3). The standard deviation for LOOCV (0.2866) was higher. This variation led to greater fluctuations in accuracy estimates during final averaging. However, the minimal difference in accuracy (0.45%) between the two methods proved the consistency and robustness of the RF model across different validation approaches. Confusion matrices generated from each method were examined (Figure 3). Minor variations were observed between them; thus, the model maintained stable predictive performance, and it was capable of accurately identifying chicken breeds regardless of dataset size or validation method used.

### 3.3. Loci Importance in Prediction Process

**Figure 4:**
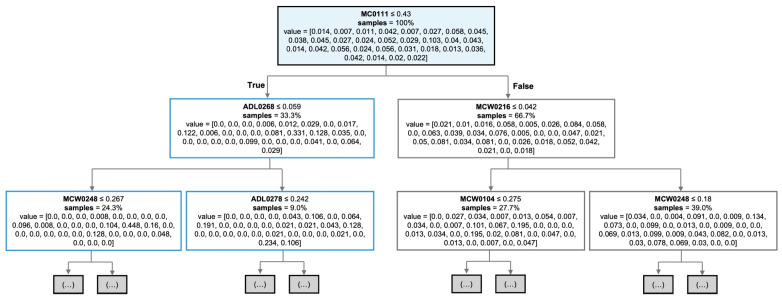
Sample of decision trees, split using entropy function. The top level shows the bootstrap sampling (random sampling) of the total dataset. The lower level shows the beginning of split using the entropy function.

The feature importance analysis was conducted using an RF model that had been trained on a fixed training set. The model identified the contribution of each locus (feature) to prediction accuracy (Agarwal et al., 2023) [66]. The analysis revealed that loci ADL0112, MCW0216, and MCW0111 had the highest importance scores, collectively contributing 20.40% of the predictive power of the model (Table S2), and the remaining 79.60% of prediction accuracy arose from other loci, highlighting the distributed nature of model performance. This finding showed the importance of retaining all loci, as their combined contributions enhanced prediction accuracy and reflected the complexity of the genotype data (Figure 4).

### 3.4. Using AutoGluon to Optimize Predictive Models in Chicken Breed Classification

**Figure 5:**
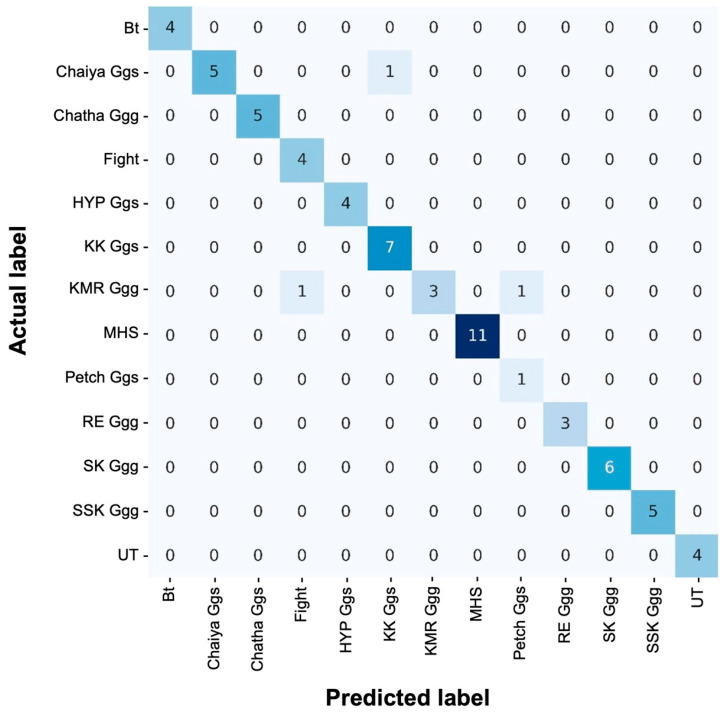
Confusion matrix of random forest trained on 85% of the total dataset and evaluated on 15% of the dataset. Darker blue shades indicate higher numbers of samples, while lighter shades represent lower counts in each cell.

The selection of the most effective predictive model for chicken breed classification was facilitated by AutoGluon, an advanced automated ML (AutoML) framework developed by Amazon Web Services. This framework automates model selection, feature engineering, hyperparameter optimization, and ensembling strategies. As an open-source AutoML library, various ML tasks, including tabular data classification, regression, image classification, object detection, and text prediction, were supported by AutoGluon [67]. Owing to its adaptability, AutoGluon is particularly well-suited for analyzing complex genetic datasets, where classification performance is influenced by intricate relationships between variables. The advantages of AutoGluon include its ability to automate model selection, allowing for the systematic evaluation of multiple ML models to identify the most suitable approach for a given dataset. We used the TabularPredictor of AutoGluon, designed for structured datasets containing both numerical and categorical variables such as genetic markers [68]. A wide range of ML algorithms, including RF, gradient boosting models (XGBoost and LightGBM), multilayer perceptrons), KNN, and SVM, were integrated into the framework [69]. These models were assessed using key performance metrics, including accuracy, precision, recall, and F1-score, which ensured that model selection was guided by empirical performance rather than arbitrary choices. Multilayer stacking, an advanced ensembling technique, was also applied to enhance predictive accuracy by combining multiple base models [70]. It involved training models independently and using a metamodel to learn from their predictions, which improved generalization and reduced overfitting. The prediction results were obtained through a robust 10-fold cross-validation strategy, ensuring reliability and generalizability (Table 5). The evaluation results showed that ensemble learning techniques, particularly WeightedEnsemble_L3 and WeightedEnsemble_L2, achieved the highest accuracy scores of 0.992 and 0.991, respectively (Table 5). These results highlight the effectiveness of stacked ensembling, where multiple base models were combined to enhance predictive performance. The superior accuracy achieved by these ensembles showed that generalization errors were reduced, improving robustness against dataset variability. Strong predictive performance was exhibited by ExtraTreesGini_BAG_L1 (0.989), NeuralNetFastAI_BAG_L2 (0.988), and LightGBMXT_BAG_L2 (0.988) (Figure 5).

**Figure 6:**
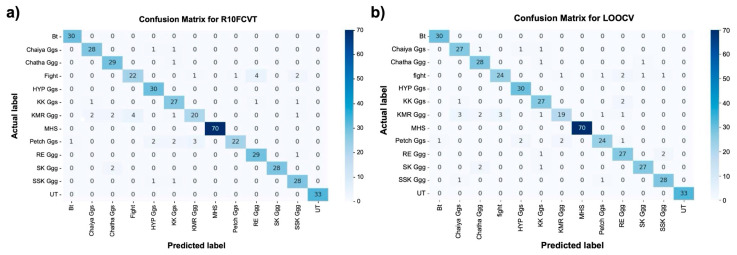
Confusion matrices of the random forest model evaluated using different cross-validation strategies. (**a**) Confusion matrix obtained from repeated 10-fold cross-validation (R10FCV), where 90% of the dataset was used for training and 10% for testing in each fold. (**b**) Confusion matrix obtained from leave-one-out cross-validation (LOOCV), in which n − 1 individuals were used for training and one individual was used for testing in each iteration. Darker blue shades indicate higher numbers of samples, while lighter shades represent lower counts in each cell.

An accuracy of 0.985 was achieved by CatBoost_BAG_L1, with a prediction time of 0.070 s. Accuracies of 0.988 were obtained by NeuralNetFastAI_BAG_L2 and LightGBMXT_BAG_L2, with fit times of 277 and 361 s, respectively. XGBoost_BAG_L1 and RandomForestGini_BAG_L1 achieved accuracies of 0.965 and 0.986. Higher accuracies of 0.992 and 0.991 were yielded by WeightedEnsemble_L3 and WeightedEnsemble_L2, respectively. Lower accuracies of 0.925 and 0.860 were obtained by KNeighborsDist_BAG_L1 and KNeighborsUnif_BAG_L1 (Figure 6).

## 4. Discussion

Breed determination is used in many applications, including authenticating mono-breed products and supporting breeding programs [71,72]. For this purpose, a variety of approaches have been proposed, including phenotypic identification, genetic testing, and pedigree analysis [73,74,75]. The growing use of computer-vision methods has been observed in animal breed confirmation, shifting from purely phenotypic human recognition to computational approaches [76,77]. In this study, the RF and AutoGluon models were used with microsatellite markers, which are frequently applied in population studies and breed classification [40,78,79]. This approach is aligned with previous studies, which have demonstrated the efficacy of this model in the domain of genetics [72,80,81,82]. The high accuracy demonstrated in this study provides further evidence of the potential of ML in breed prediction, as supported by classification tasks in related fields [83,84,85]. By contrast, AutoGluon, which automates the entire ML—including data preprocessing, feature engineering, model selection, hyperparameter tuning, and ensemble learning—is capable of training and evaluating diverse models, such as tree-based methods, linear models, neural networks, and KNN. These models were combined through advanced stacking techniques to enhance performance. Consequently, AutoGluon provides a more flexible, automated, and potentially more accurate approach compared to the standalone RF model.

### 4.1. Performance Metrics, Impurity Criteria, and Marker Optimization for RF Models

The F1-score, which ranges between 0 and 1 with 1 indicating perfect classification, was used as a performance measure [86]. The high macro average F1-score observed reflects an effective balance between precision and recall across all classes, demonstrating that the algorithm performs robustly and effectively handles diverse class distributions in this classification task [60]. Entropy and Gini—widely used impurity measures in decision-tree algorithms—typically produce comparable results [87]. While Gini is more computationally straightforward, entropy provides a more nuanced measure of information gain, which can lead to slightly improved performance in complex classification tasks [61,88]. The use of entropy in this study aligns with the goal of maximizing model accuracy, demonstrating its practical classification superiority. R10FCV is considered the best evaluation method, despite the similar performance observed with LOOCV [89,90]. The results presented here support this, showing slight performance improvement and greater stability with R10FCV. LOOCV is time-consuming during training [90]. Another frequently used evaluation technique with an equivalent objective is the out-of-bag (OOB) estimate [91]. In contrast to cross-validation, which explicitly divides the dataset into training and testing subsets (folds), the OOB method provides internal validation during training by using approximately 37% of each bootstrap sample [92]. Both OOB prediction and cross-validation can be used to evaluate classifier performance, providing robust estimates. However, studies have shown that cross-validation is generally preferred over OOB because it better facilitates the selection of optimal hyperparameters, such as m_try_ in RF models [93,94]. This preference is especially important when fine-tuning model performance, as cross-validation allows more explicit control over data splitting and evaluation. Rasoarahona et al. (2023) [95] proposed an efficient method for selecting a reduced set of microsatellite panels that can be used similarly to the full list for population characterization and origin determination. This methodology demonstrated its efficacy for individual identification in certain animal breeds [96,97,98]. Applying this method in future experiments with chicken data, within the current RF approach, will enable the RF model to operate with fewer independent features. The performance of the resulting model will be evaluated to determine whether it remains comparable to or surpasses the current performance.

### 4.2. Model Performance, Computational Considerations, and Locus Importance

AutoGluon was applied to optimize predictive models for chicken breed classification, demonstrating its effectiveness in automating model selection and hyperparameter tuning. The evaluation metrics showed that ensemble techniques, particularly WeightedEnsemble_L3 and WeightedEnsemble_L2, achieved the highest accuracy scores of 0.992 and 0.991, respectively, highlighting the strength of stacked ensembling in improving predictive performance. The superior accuracy of these ensembles suggests that the aggregation of diverse models reduces generalization errors and enhances robustness against data variability. Of the individual models, ExtraTreesGini_BAG_L1 (0.989), NeuralNetFastAI_BAG_L2 (0.988), and LightGBMXT_BAG_L2 (0.988) demonstrated strong predictive performance. The presence of ExtraTrees algorithms among the top models aligns with existing reports of their efficiency in high-dimensional, non-linear data [99]. NeuralNetFastAI models, which are based on deep-learning architectures, and LightGBM models, which are known for their effectiveness in structured data, also achieved high accuracy, reinforcing their utility in breed classification [26]. From a computational efficiency perspective, this experiment was conducted on an Apple M1 system. CatBoost_BAG_L1 achieved 0.985 accuracy with a prediction time of 0.070 s, indicating its suitability for real-time inference. NeuralNetFastAI_BAG_L2 and LightGBMXT_BAG_L2, despite high accuracy, had significantly longer fit times of 277 and 361 s, respectively. This demonstrates a trade-off between model performance and computational cost, which is important in large-scale applications with limited resources. Models such as XGBoost_BAG_L1 (0.965 accuracy) and RandomForestGini_BAG_L1 (0.986 accuracy) performed well, but their longer fit times suggest that ensemble methods like WeightedEnsemble_L3 offer a better balance between accuracy and training efficiency. KNN models (KNeighborsDist_BAG_L1: 0.925, KNeighborsUnif_BAG_L1: 0.860) demonstrated lower accuracy, which highlights their limitations in handling high-dimensional genetic datasets due to their sensitivity to noise and lack of feature selection mechanisms. The complexity of the dataset, characterized by 28 distinct bi-allelic loci, posed a challenge during model training because effective learning requires a substantial number of samples [100,101]. The limited genotype data for certain populations restricted the scope of analysis, indicating that more extensive datasets are necessary in future studies. The stochastic nature of RF algorithms poses a challenge in identifying key loci. However, this method had a significant advantage of RF over alternative ML methods and has been effective in other studies in identifying informative loci panels [28,102,103,104,105]. Future studies should compare multiple classification methods across different tasks to determine the most informative loci for chicken breed prediction.

### 4.3. Computational Trade-Offs in Model Selection

While ensemble and deep-learning models achieved the highest predictive accuracies, the associated computational costs varied significantly [106]. Although LightGBMXT_BAG_L2 and NeuralNetFastAI_BAG_L2 demonstrated strong classification performance, their long-fit times may limit their practicality in time-sensitive or resource-constrained settings [68]. In contrast, CatBoost_BAG_L1 offered a favorable balance between accuracy and computational efficiency, making it suitable for real-time inference. Therefore, both predictive performance and resource requirements should be considered when selecting models for large-scale genetic classification tasks, especially in field applications or under limited computational infrastructure [107].

### 4.4. Contributions to the Identification and Conservation of Breeds

Effective breed recognition is crucial for breeding program success and conservation of genetic resources, but it remains challenging, especially for non-experts [108,109,110]. While SNP-based ML models have shown promise in breed classification [72,111], this study explores an alternative method using microsatellite genotypes, which may offer higher informativeness per genetic locus in certain species, such as chicken, and may be less costly if fewer samples are tested [95]. Although image recognition is a modern phenotypic classification method, it often depends on high-quality images and preprocessing [112]. This study proposes a novel ML-based approach that uses the correlation between microsatellite markers and population-specific traits. The results demonstrate that methods like RF can effectively support breed identification, enriching tools available in animal genetics. However, the accuracy of ML-based approaches using genetic data is still limited by the small genotypic library of animal breeds, which does not represent the full genetic diversity of chickens. A large genotypic library with many individuals across different localities is necessary to improve the reliability of breed identification.

## 5. Conclusions

**Figure 7:**
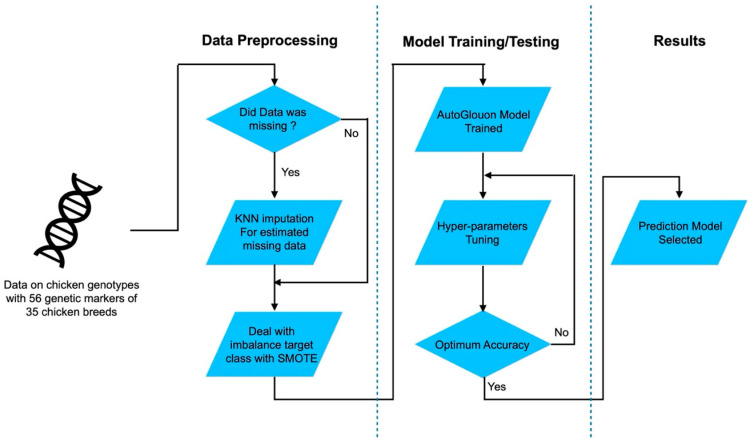
The workflow outlines data preprocessing (handling missing data with k-nearest neighbors imputation and class imbalance with synthetic minority oversampling technique), model training and evaluation using AutoGluon with hyperparameter tuning, and selection of the optimal prediction model.

This study investigated the effectiveness of the RF model for chicken breed classification using microsatellite genotypes, and strong performance was demonstrated across multiple metrics (Figure 7). The accuracy of the model was significantly improved by AutoGluon’s automated ensembling, which outperformed traditional models. Weighted ensembles, particularly those combining ExtraTrees, LightGBM, and deep-learning models, achieved the best results, especially for complex genetic data. However, computational time remains a concern for high-accuracy models. These findings highlight the potential of AutoGluon as an AutoML tool for genetic classification, offering automation and robust performance for biological and agricultural research. Future efforts should focus on optimizing computational efficiency to enable real-time applications. Additionally, future studies could integrate multi-omics data, including approaches applied in recent single-cell transcriptomic analyses of avian performance [113,114], to further enhance the predictive accuracy and biological interpretability of breed classification models.

## Figures and Tables

**Table 1:** Distribution of chicken population genotype data after exclusion of less than 30 individuals, and random split from the dataset division into 85% training and 15% testing data.

Population	Aberrations	Number of Individuals	Training Dataset	Testing Dataset
Betong	Bt	30	26	4
Chaiyaphum (*G. g. spadiceus*)	Chaiya Ggs	30	24	6
Chaiyaphum (*G. g. gallus*)	Chatha Ggg	30	25	5
Fighting chicken	fight	30	26	4
Huai Yang Pan (*G. g. spadiceus*)	HYP Ggs	30	26	4
Khao Kho (*G. g. spadiceus*)	KK Ggs	30	23	7
Khok Mai Rua (*G. g. gallus*)	KMR Ggg	30	25	5
Mae Hong Son	MHS	70	59	11
Petchaburi (*G. g. spadiceus*)	Petch Ggs	30	29	1
Roi Et (*G. g. gullus*)	RE Ggg	30	27	3
Sa Kaeo (*G. g. gullus*)	SK Ggg	30	24	6
Si Sa Ket (*G. g. gullus*)	SSK Ggg	30	25	5
Uthai Thani (Samae Dam)	UT	33	29	4
Total		433	368	65

**Table 2:** Functionality of the random forest decision according to the probability of class membership. Illustrations of 10 prediction cases among the testing data processed with the trained model.

True Label	First Class		Second Class		Third Class		Final Prediction
	Membership Probability	Population	Membership Probability	Population	Membership Probability	Population	
KK Ggs	52.81	KK Ggs	14.16	KMR Ggg	7.64	SK Ggg	KK Ggs
MHS	97.98	MHS	0.90	UT	0.45	KMR Ggg	MHS
SK Ggg	44.72	SK Ggg	15.96	Chaiya Ggs	10.56	Chatha Ggg	SK Ggg
fight	36.40	fight	13.71	KMR Ggg	12.36	SSK Ggg	fight
KMR Ggg	16.40	fight	14.16	Petch Ggs	12.58	Chatha Ggg	fight
fight	50.34	fight	13.71	RE Ggg	8.76	KMR Ggg	fight
MHS	92.81	MHS	3.60	UT	0.90	Chatha Ggg	MHS
SSK Ggg	48.54	SSK Ggg	19.33	RE Ggg	17.53	fight	SSK Ggg
fight	26.97	fight	22.47	KMR Ggg	13.93	SSK Ggg	fight
Chaiya Ggs	63.60	Chaiya Ggs	10.11	KK Ggs	8.76	KMR Ggg	Chaiya Ggs

**Table 3:** Performance assessments through overall accuracy, 95% confidence interval, Cohen’s Kappa value, and No-information rate after training random forest models across data validation techniques.

Method	Accuracy (%)	Accuracy Std	95% CI	Kappa	NIR
Fixed data split	95.38	-	(0.9028, 1.0000)	0.9492	0.1692
R10FCVT	91.44	0.0408	(0.8904, 0.9384)	0.9065	0.1617
LOOCV	90.99	0.2866	(0.8830, 0.9369)	0.9016	0.1617

**Table 4:** Classification report showing precision, recall, and F1-score, with a random forest model after hyperparameter tuning and training with fixed data splits.

Population	Precision	Recall	F1-Score
Bt	1.00	1.00	1.00
Chaiya Ggs	1.00	0.83	0.91
Chatha Ggg	1.00	1.00	1.00
fight	0.80	1.00	0.89
HYP Ggs	1.00	1.00	1.00
KK Ggs	0.88	1.00	0.93
KMR Ggg	1.00	0.60	0.75
MHS	1.00	1.00	1.00
Petch Ggs	0.50	1.00	0.67
RE Ggg	1.00	1.00	1.00
SK Ggg	1.00	1.00	1.00
SSK Ggg	1.00	1.00	1.00
UT	1.00	1.00	1.00
macro average	0.94	0.96	0.93
weighted average	0.97	0.95	0.95

**Table 5:** Performance Evaluation of AutoGluon Models for Chicken Breed Classification.

Model	Score (Accuracy)	Prediction Time (s)	Fit Time (s)	Pred Time Marginal (s)	Fit Time Marginal (s)	Stack Level	Fit Order
WeightedEnsemble_L3	0.992000	3.831986	277.421132	0.000882	0.280986	3	17
WeightedEnsemble_L2	0.991429	0.367359	2.854856	0.000486	0.125387	2	13
ExtraTreesGini_BAG_L1	0.989143	0.110687	1.107739	0.110687	1.107739	1	9
NeuralNetFastAI_BAG_L2	0.988571	3.831105	277.140146	0.204727	7.725666	2	14
ExtraTreesEntr_BAG_L1	0.988000	0.163950	0.799778	0.163950	0.799778	1	10
LightGBMXT_BAG_L2	0.988000	4.605415	361.044465	0.979038	91.629985	2	15
LightGBMXT_BAG_L1	0.987429	1.184527	14.656198	1.184527	14.656198	1	4
RandomForestGini_BAG_L1	0.986286	0.086773	0.875183	0.086773	0.875183	1	6
LightGBM_BAG_L2	0.986286	3.823396	302.657472	0.197019	33.242992	2	16
CatBoost_BAG_L1	0.985714	0.070553	189.957427	0.070553	189.957427	1	8
RandomForestEntr_BAG_L1	0.985143	0.092236	0.821953	0.092236	0.821953	1	7
NeuralNetFastAI_BAG_L1	0.972571	0.072332	4.908889	0.072332	4.908889	1	3
NeuralNetTorch_BAG_L1	0.970857	0.174236	27.011140	0.174236	27.011140	1	12
LightGBM_BAG_L1	0.970857	1.020726	19.746374	1.020726	19.746374	1	5
XGBoost_BAG_L1	0.965714	0.387963	9.517267	0.387963	9.517267	1	11
KNeighborsDist_BAG_L1	0.925143	0.109600	0.003412	0.109600	0.003412	1	2
KNeighborsUnif_BAG_L1	0.860571	0.152794	0.009120	0.152794	0.009120	1	1

Parameter Descriptions: Model: The machine learning model used for classification. Score (Accuracy): The classification accuracy of the model. Prediction Time (s): Time taken to generate predictions. Fit Time (s): Time taken to train the model. Pred Time Marginal (s): Additional time taken for predictions compared to previous models. Fit Time Marginal (s): Additional time required for model training. Stack Level: The level at which the model is stacked in the ensemble process. Fit Order: The order in which the model was fitted during the AutoGluon pipeline.

## Data Availability

The original contributions presented in this study are included in the article. Further inquiries can be directed to the corresponding authors.

## Supplementary Materials

The following supporting information can be downloaded at https://www.mdpi.com/article/10.3390/biology15010021/s1, Table S1: Initial materials including 30 populations with number of available individuals. Table S2: Ranking of 28 loci importances in the breed prediction made with the random forest model built with a fixed training dataset. Figure S1: Correlations between predicted and observed breed classifications used as additional accuracy metrics. References [10,20,41,42,115,116,117,118,119,120,121,122] are cited in the supplementary materials.
