# Supplementary material for: High-Accuracy Chicken Breed Identification Using Microsatellite Genotype Data and AutoGluon Framework
Source: Biology (Basel). 2025 Dec 22;15(1):21. doi: 10.3390/biology15010021 (PMC12785061; doi:10.3390/biology15010021)
Supplement: Supplementary file 1 [file biology-15-00021-s001.zip › biology-4034829-supplementary.pdf]

Supplementary Information

High-accuracy chicken breed identification using microsatellite genotype data and AutoGluon framework by Toky et al.

Analysis pipeline and code

The hyperparameters used in the final optimized models for all methods employed. The hyperparameters of the final models are as follows:

```
'RF': {'n_estimators': [100, 200], 'max_depth': [5, 10, 15], 'min_samples_split': [2, 5],
'min_samples_leaf': [1, 2]}

'NN_TORCH': [{}, {'activation': 'elu', 'dropout_prob': 0.10077639529843717, 'hidden_size': 108,
'learning_rate': 0.002735937344002146, 'num_layers': 4, 'use_batchnorm': True, 'weight_decay':
1.356433327634438e-12, 'ag_args': {'name_suffix': '_r79', 'priority': -2}}, {'activation': 'elu',
'dropout_prob': 0.11897478034205347, 'hidden_size': 213, 'learning_rate':
0.0010474382260641949, 'num_layers': 4, 'use_batchnorm': False, 'weight_decay':
5.594471067786272e-10, 'ag_args': {'name_suffix': '_r22', 'priority': -7}}],

'GBM': [{'extra_trees': True, 'ag_args': {'name_suffix': 'XT'}}, {}, 'GBMLarge'],

'CAT': [{}, {'depth': 6, 'grow_policy': 'SymmetricTree', 'l2_leaf_reg': 2.1542798306067823,
'learning_rate': 0.06864209415792857, 'max_ctr_complexity': 4, 'one_hot_max_size': 10,
'ag_args': {'name_suffix': '_r177', 'priority': -1}}, {'depth': 8, 'grow_policy': 'Depthwise',
'l2_leaf_reg': 2.7997999596449104, 'learning_rate': 0.031375015734637225, 'max_ctr_complexity':
2, 'one_hot_max_size': 3, 'ag_args': {'name_suffix': '_r9', 'priority': -5}}],

'XGB': [{}, {'colsample_bytree': 0.6917311125174739, 'enable_categorical': False, 'learning_rate':
0.018063876087523967, 'max_depth': 10, 'min_child_weight': 0.6028633586934382, 'ag_args':
{'name_suffix': '_r33', 'priority': -8}}, {'colsample_bytree': 0.6628423832084077,
'enable_categorical': False, 'learning_rate': 0.08775715546881824, 'max_depth': 5,
'min_child_weight': 0.6294123374222513, 'ag_args': {'name_suffix': '_r89', 'priority': -16}}],

'FASTAI': [{}, {'bs': 256, 'emb_drop': 0.5411770367537934, 'epochs': 43, 'layers': [800, 400], 'lr':
0.01519848858318159, 'ps': 0.23782946566604385, 'ag_args': {'name_suffix': '_r191', 'priority': -
4}}, {'bs': 2048, 'emb_drop': 0.05070411322605811, 'epochs': 29, 'layers': [200, 100], 'lr':
0.08974235041576624, 'ps': 0.10393466140748028, 'ag_args': {'name_suffix': '_r102', 'priority': -
11}}],

'XT': [{'criterion': 'gini', 'ag_args': {'name_suffix': 'Gini', 'problem_types': ['binary',
'multiclass']}}, {'criterion': 'entropy', 'ag_args': {'name_suffix': 'Entr', 'problem_types': ['binary',
'multiclass']}}, {'criterion': 'squared_error', 'ag_args': {'name_suffix': 'MSE', 'problem_types':
['regression', 'quantile']}}],

'KNN': [{'weights': 'uniform', 'ag_args': {'name_suffix': 'Unif'}}, {'weights': 'distance', 'ag_args':
{'name_suffix': 'Dist'}}],

NeuralNetwork = Sequential([
    Dense(128, activation='relu', input_shape=(X_train.shape[1],)),
    Dropout(0.3),
```

```
Dense(64, activation='relu'),
Dropout(0.3),
Dense(32, activation='relu'),
Dense(len(label_encoder.classes_), activation='softmax')
```

The full analysis pipeline and code have been provided to ensure reproducibility. The Jupyter Notebook is available at the following links:

1) https://colab.research.google.com/drive/1YVWFbAtbVtaOYpXdR3ShgMAukhwJuBpS?usp=sharing
2) https://colab.research.google.com/drive/1uQcQRfA0n_dcYHE0C1E-DkJPEgwsvZ_3?usp=sharing

Supplementary Tables

Table S1. Initial materials including 30 populations with number of available individuals.

No.	Population Aberrations	Number of Samples	Population	Characteristics and Genetic Background
1	ChaingR Ggg	9	Chiang Rai (*G. g. gallus*)	Red Junglefowl widely distributed in Thailand comprise two subspecies, *Gallus gallus spadiceus* and *G. g. gallus*, which retain typical wild-type characteristics of junglefowl. In Thailand, these birds are conserved and selectively bred under managed programs at breeding and conservation centers operated by the Department of National Parks, Wildlife and Plant Conservation, Thailand, and the Zoological Park Organization of Thailand. [1,2]
2	Chaiya Ggs	30	Chaiyaphum (*G. g. spadiceus*)	
3	Chantha Ggg	30	Chaiyaphum (*G. g. gallus*)	
4	HS Ggg	4	Huai Sai (*G. g. gallus*)	
5	HS Ggs	15	Huai Sai (*G. g. spadiceus*)	
6	HYP Ggs	30	Huai Yang Pan (*G. g. spadiceus*)	
7	KK Ggs	30	Khao Kho (*G. g. spadiceus*)	
8	KKZ	19	Khon Kaen Zoo	
9	KMR Ggg	30	Khok Mai Rua (*G. g. gallus*)	
10	Petch Ggs	30	Petchaburi (*G. g. spadiceus*)	
11	RE Ggg	30	Roi Et (*G. g. gullus*)	
12	SK Ggg	30	Sa Kaeo (*G. g. gullus*)	
13	SSK Ggg	30	Si Sa Ket (*G. g. gullus*)	
14	SKZ	16	Songkhla Zoo	
15	CRZ	19	Chiang Rai Zoo	
16	CMZ	7	Chiang Mai Zoo	
17	BLBF	10	Nin Kaset (black)	Thai native black-meat chicken developed by Kasetsart University, valued for its high-quality eggs [1–3]
18	BLWF	10	Nin Kaset (white)	
19	BT	30	Betong	Betong chicken is a traditional local chicken breed from Betong district, Yala Province, Thailand, well known for its distinctive flavor and long-standing culinary heritage [1,2,4]
20	CH	10	Chee	Thai native chicken breed characterized by its entirely white plumage, resembling the appearance of a Buddhist nun, from which its name is derived. It is hardy, disease-resistant, easy to raise, and suitable for both meat and egg production [1,2]
21	Cross	6	Cross bred	Crossbred chickens result from crossing different breeds, leading to a mixed genetic background that enhances productivity and performance [5]

22	DT	5	Dong-Tao	Dong Tao chicken is a native Vietnamese breed from northern Vietnam, renowned for its premium meat quality, high consumer demand, and limited availability, which contribute to its exceptionally high market value [6]
23	fight	30	Fighting chicken	Thai fighting chickens hold significant cultural importance, with genetic analyses indicating strong directional selection and admixture with domestic breeds, while showing limited genetic influence from red junglefowl. [7,8]
24	KP	10	Kheaw-Paree	An indigenous Thai chicken breed characterized by its distinctive blackish-green appearance and well-defined morphological traits. This breed is important for local genetic conservation, breed improvement, and supporting rural livelihoods through sustainable poultry production [7,9]
25	LHK	17	Lueng-hang-khao	Lueng Hang Khao is a distinctive native chicken breed with a long cultural history in Phitsanulok Province, Thailand [10]
26	LPK	22	Lao Pa Koi	A popular Thai fighting breed shaped by selective breeding and serves as a useful model for studying genetic diversity and admixture between red junglefowl and domestic chickens [7].
27	MHS	70	Mae Hong Son	A native domestic chicken breed from northern Thailand, widely distributed in Mae Hong Son Province. Genetic analyses reveal introgression from red junglefowl and other indigenous breeds, supporting its origin as a crossbreed between red junglefowl and Thai village chickens adapted to local environmental, social, and cultural conditions [11].
28	NP	29	Nakhon Pathom (Ornamental chickens)	Ornamental chickens are chicken breeds selectively developed for beauty and appearance rather than for meat or egg production [7].
29	PHD	10	Pradu-hang-dam	An indigenous Thai chicken breed valued for its good growth performance, flavorful meat, and attractive appearance. It is characterized by black tail feathers, red-mixed body plumage, a red-and-black face, and a pea comb [12].
30	UT	33	Uthai Thani (Samae Dam)	A traditional black-skinned chicken breed from Uthai Thani province, distinguished by its entirely black appearance, including skin, bones, and feathers. Although closely related to PDH, SD chickens are often regarded by local communities as a distinct breed due to their unique morphology and cultural significance [12].

Table S2. Ranking of 28 loci importances in the breed prediction made with the random forest model built with fixed training dataset.

Locus	Importance in prediction (%)
ADL0112	6.8971
MCW0216	6.8891
MCW0111	6.6091
MCW0248	5.9539
MCW0330	5.6968
MCW0078	5.0678
LEI0234	4.7788
MCW0165	4.5701
MCW0104	4.0529
ADL0278	3.9660
MCW0183	3.8971
MCW0034	3.8934
LEI0192	3.7571
MCW0037	3.3460
ADL0268	3.2593
MCW0016	3.2138
MCW0222	2.8389
MCW0069	2.6756
MCW0206	2.6344
MCW0295	2.6079
LEI0094	2.2495
MCW0081	2.1610
MCW0123	1.9117
MCW0014	1.9006
MCW0103	1.7911
MCW0067	1.4493
LEI0166	1.3596
MCW0098	0.5721

Supplementary Figure

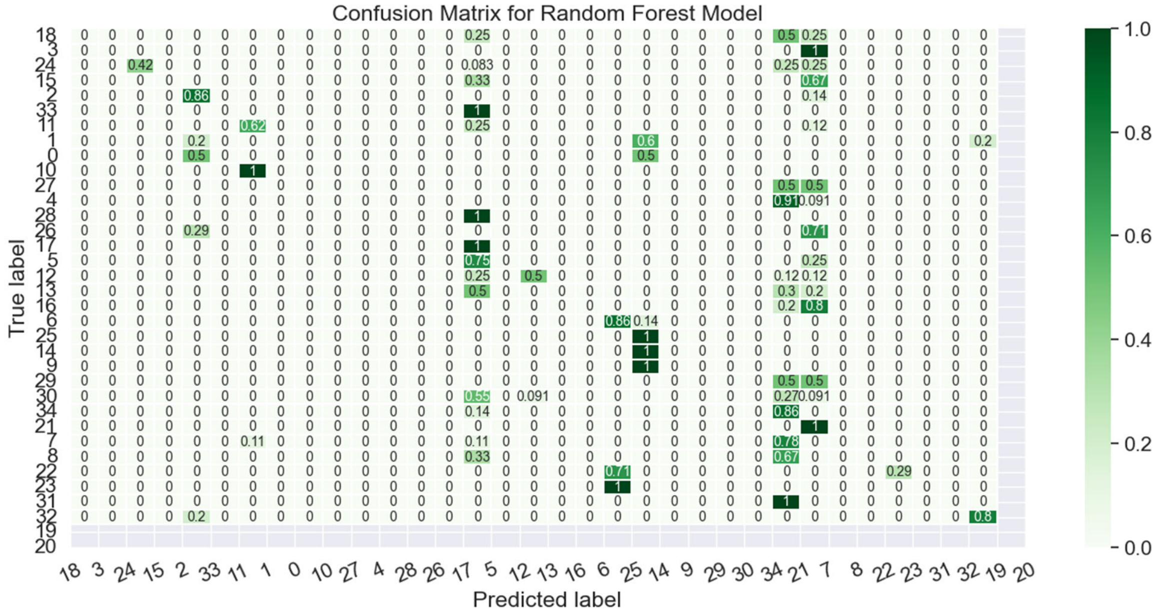

Figure S1. Correlations between predicted and observed breed classifications used as additional accuracy metrics.

References

1) Hata, A.; Nunome, M.; Suwanasopee, T.; Duengkae, P.; Chaiwatana, S.; Chamchumroon, W.; Suzuki, T.; Koonawootrittriron, S.; Matsuda, Y.; Srikulnath, K. Origin and evolutionary history of domestic chickens inferred from a large population study of Thai red junglefowl and indigenous chickens. *Scientific reports* **2021**, *11*, 2035.
2) Singchat, W.; Chaiyes, A.; Wongloet, W.; Ariyaraphong, N.; Jaisamut, K.; Panthum, T.; Ahmad, S.F.; Chaleekarn, W.; Suksavate, W.; Inpota, M. Red junglefowl resource management guide: Bioresource reintroduction for sustainable food security in Thailand. *Sustainability* **2022**, *14*, 7895.
3) Munkong, P.; Suwanasopee, T.; Koonawoottrittriron, S. Morphometric Analysis of Kai Dam Nil Kaset (Sart) Eggs: Implications for Production and Selection. *Khon Kaen Agriculture Journal*, **Supplement** (Agricultural Conference), Khon Kaen University, Thailand.
4) Maneechot, N.; Tunim, S.; Wattanachant, C.; Khongsen, M.; Sukteab, P.; Phongphanich, P. Genetic Diversity of Native Chicken Populations and Red Jungle Fowl in Southern Thailand Based on Mitochondrial DNA D-loop Region. *Brazilian Journal of Poultry Science* **2025**, *27*, 001–010.
5) Mancinelli, A.C.; Menchetti, L.; Birolo, M.; Bittante, G.; Chiattelli, D.; Castellini, C. Crossbreeding to Improve Local Chicken Breeds: Predicting Growth Performance of the Crosses Using the Gompertz Model and Estimated Heterosis. *Poultry Science* **2023**, *102*, 102783.
6) Siddiqui, S.A.; Rahmatullah, R.A.; Achyar, A.; Atifah, Y.; Ahmad, A.; Fitriani, A. Dong Tao Chickens in Vietnam—A Critical Review. *World's Poultry Science Journal* **2024**, *80*, 1241–1263.
7) Wattanadilokcahtkun, P.; Chalermwong, P.; Singchat, W.; Wongloet, W.; Chaiyes, A.; Tanglertpaibul, N.; Budi, T.; Panthum, T.; Ariyaraphong, N.; Ahmad, S.F. Genetic admixture and diversity in Thai domestic chickens revealed through analysis of Lao Pa Koi fighting cocks. *Plos one* **2023**, *18*, e0289983.
8) Budi, T.; Luu, A.H.; Singchat, W.; Wongloet, W.; Rey, J.; Kumnan, N.; Chalermwong, P.; Nguyen, C.P.T.; Panthum, T.; Tanglertpaibul, N.; Thong, T.; Ali, H.; Vangnai, K.; Chaiyes, A.; Yokthongwattana, C.; Sinthuvanich, C.; Han, K.; Antunes, A.; Muangmai, N.; Duengkae, P.; Srikulnath, K. Purposive Breeding Strategies Drive Genetic Differentiation in Thai Fighting Cock Breeds. *Genes & Genomics* **2024**, *46*, 1225–1237.
9) Phromnoi, S.; Yeamkong, S.; Mingchai, C. Phenotypic Characteristics and Morphology of Khiew-Phalee Chicken in Uttaradit Province. *Rajamangala University of Technology Srivijaya Research Journal* **2023**, *15*(1), 37–48.
10) Somkuna, E.; Intaravicha, N.; Maksuwan, A. Effects of Natural Environmental Structure on Growth Performance of Indigenous Chicks (Lueng Hang Khao). *Journal of Vocational Education in Agriculture* **2023**, *6*(2), 68–83.
11) Wongloet, W.; Singchat, W.; Chaiyes, A.; Ali, H.; Piangporntip, S.; Ariyaraphong, N.; Budi, T.; Thienpreecha, W.; Wannakan, W.; Mungmee, A.; Jaisamut, K.; Thong, T.; Panthum, T.; Ahmad, S.F.; Lisachov, A.; Suksavate, W.; Muangmai, N.; Chuenka, R.; Nunome, M.; Chamchumroon, W.; Han, K.; Nuangmek, A.; Matsuda, Y.; Duengkae, P.; Srikulnath, K. Environmental and Socio-Cultural Factors Impacting the Unique Gene Pool Pattern of Mae Hong Son Chicken. *Animals* **2023**, *13*, 1949.
12) Tanglertpaibul, N.; Budi, T.; Nguyen, C.P.T.; Singchat, W.; Wongloet, W.; Kumnan, N.; Chalermwong, P.; Luu, A.H.; Noito, K.; Panthum, T. Samae Dam chicken: A variety of the Pradu Hang Dam breed revealed from microsatellite genotyping data. *Anim. Biosci.* **2024**, *37*, 2033.
